# Uretero-Pelvic Junction Stenosis: Considerations on the Appropriate Timing of Correction Based on an Infant Population Treated with a Minimally-Invasive Technique

**DOI:** 10.3390/children8020107

**Published:** 2021-02-04

**Authors:** Mario Lima, Niel Di Salvo, Andrea Portoraro, Michela Maffi, Giovanni Parente, Vincenzo Davide Catania, Tommaso Gargano

**Affiliations:** Pediatric Surgery Unit, IRCCS Sant’Orsola-Malpighi Hospital, University of Bologna, 40138 Bologna, Italy; mario.lima@unibo.it (M.L.); andrea.portoraro@hotmail.it (A.P.); michela.maffi@aosp.bo.it (M.M.); giovanni.parente@outlook.com (G.P.); vincenzodavide.catania@aosp.bo.it (V.D.C.); tommaso.gargano2@unibo.it (T.G.)

**Keywords:** uretero-pelvic junction obstructions (UPJO), hydronephrosis, pyeloplasty

## Abstract

There is no univocal consensus about timing of intervention and best surgical approach for infants with asymptomatic uretero-pelvic junction obstruction (UPJO). We conducted a retrospective analysis of patients undergoing one-trocar-assisted pyeloplasty (OTAP) in a 13 year range period by creating two homogenous groups (indications for surgery were the same for all patients): patients operated on in the first 90 days of life (34 patients; Group 1) and patients operated on between 3 and 12 months of life (34 patients; Group 2). We observed no statistically significant differences between groups in regard to mean operative time, conversion rate to open surgery, mean hospital stay, early complications (urinary leakage) rate and mean antero-posterior diameter (APD) reduction rate. Moreover, no statistical improvement was seen between groups in regard to separate renal function (SRF) at 1-year-follow-up renogram. Thanks to the HSS calculated before and 1 year after surgery, we registered an important improvement in Group I patients (*p* = 0.023). In our study, there was no significant evidence, in terms of intraoperative data and early postoperative outcomes, between patients who underwent an early pyeloplasty and those who underwent a delayed correction. Nevertheless, we registered a significant improvement in those patients with an impaired SRF that underwent an early surgical correction, especially in terms of urinary flow. Even though this study cannot definitely establish the superiority of early timing of correction, it is evident that further research is needed to clarify this aspect.

## 1. Introduction

Uretero-pelvic junction obstruction (UPJO) is the most common cause of prenatal hydronephrosis [1,2,3].

Unfortunately, to date, despite the presence of a huge number of articles in the international literature, there is no univocal consensus about timing of correction and the best surgical approach for infants. Some authors believe in the importance of an early intervention to preserve renal function; others think a delayed correction, apart from/beyond being less risky and technically easier, does not affect renal parenchymal integrity [4,5,6,7].

Since 2005, in our institution, the preferred approach for hydronephrosis in children younger than 2 years has been the one-trocar-assisted pyeloplasty (OTAP) [8,9]. This procedure combines the advantages of a minimally invasive technique with the high success rate of standard dismembered pyeloplasty; it had already been proven to be a feasible technique in terms of efficacy and safety in patients treated in the first 90 days of life [10].

With the present study, we wanted to compare patients who underwent an early pyeloplasty (in the first 90 days of life) with patients who underwent a delayed pyeloplasty (3–12 months) in terms of outcomes and hydronephrosis’ improvement between 1 and 3 years after UPJO correction.

## 2. Materials and Methods

We retrospectively reviewed all charts of infants treated with OTAP for UPJO at our institution between January 2005 and September 2018.

Informed consent to use these data was given by the parents of each single patient with approval of our University Hospital’s Institutional Review Board with the following ethical committee approval code: CHPED-APR-19-20.

We then selected and examined the ones, born on term, with a diagnosis of severe hydronephrosis made in the first 60 days of life. Premature babies were then excluded from the analysis. All of them presented a prenatal suspicion of hydronephrosis. Suspicion was then confirmed by ultrasounds in the first days of life and repeated after two weeks. Voiding cystourethrogram (VCUG) and sequential renogram with MAG-3 and furosemide injection were carried out respectively at 2 weeks and 1 month of life. Ultrasounds and VCUG were only performed by 4 experienced pediatric radiologists, experts in urinary tract malformations.

Indications for surgical correction were the same for all patients and were the following:

Antero-posterior pelvic diameter (APD) > 20 mm and an obstructive pattern on MAG3 with furosemide, associated with an impaired echotexture and/or separate renal function (SRF) < 40%. For impaired echotexture we meant a reduction of parenchymal thickness and/or lack of cortico-medullary differentiation and/or flattening of papillae. The above ultrasonographic features are the ones included in the Society of Fetal Urology’s (SFU) grading system of hydronephrosis.

Thanks to these criteria, we were sure to operate on actual stenosis, as confirmed by all histological reports. At our institution, patients that do not fulfill these criteria are re-evaluated after 3–6 months with ultrasounds and/or second renography, but these were not considered in our analysis (excluded).

Furthermore, we divided patients into two different groups: the first included patients operated on in the first 90 days of life (Group 1), and the second one included those operated on after 90 days of life and within the first year of life (Group 2). The choice to operate early or after was explained, shared and made with parents, after clear explanation of the unsolved issue, that is to say, the lack of consensus about the timing of the intervention.

### Description of the Technique

With the patient placed in lateral decubitus on the non-pathologic side, exposing the pathologic flank, a 12 mm long incision is made on the prolongation of the 11th to 12th rib. The Gerota fascia and the perirenal fat are reached anteriorly after blunt dissection through the muscles. A 10 mm balloon anchorage trocar is inserted and we use a 10 mm 0° lens operative telescope with a 5 mm operative channel.

**Figure 1:**
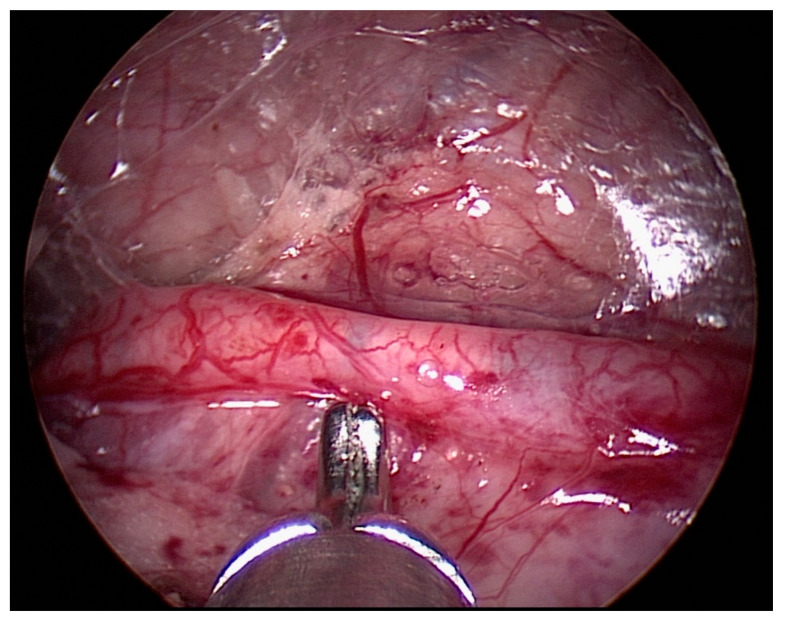
Ureter identification during the retroperitoneoscopic phase. The L dissector, inserted in the operative channel of the operative telescope, can be seen.

**Figure 2:**
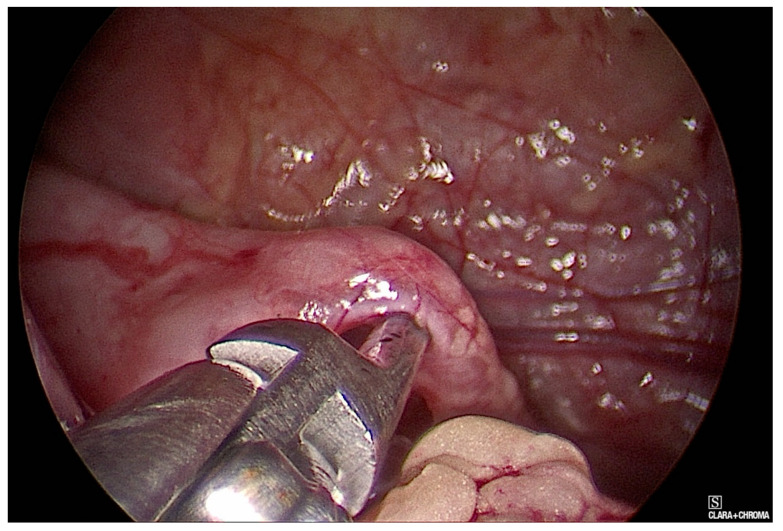
Isolation of the Uretero-Pelvic Junction (UPJ) with the L dissector.

**Figure 3:**
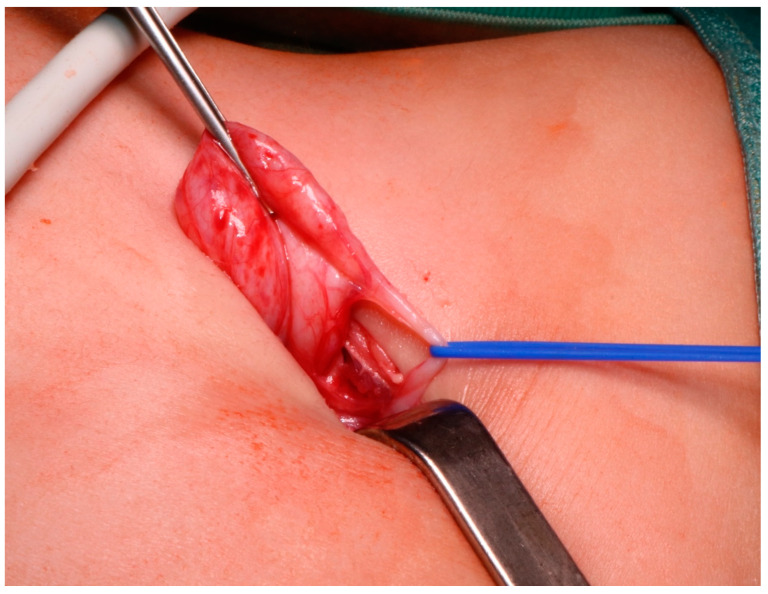
The UPJ is brought out through the lumbar incision using a vessel loop, inserted during the retroperitoneoscopic phase.

**Figure 4:**
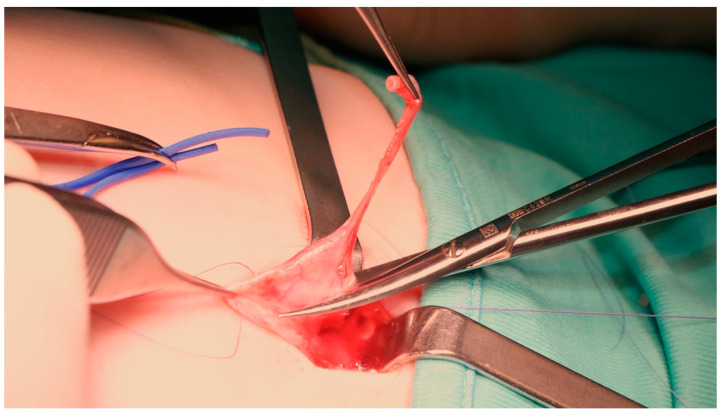
The obstructed UPJ with part of the dilated pelvis and proximal ureter are removed.

**Figure 5:**
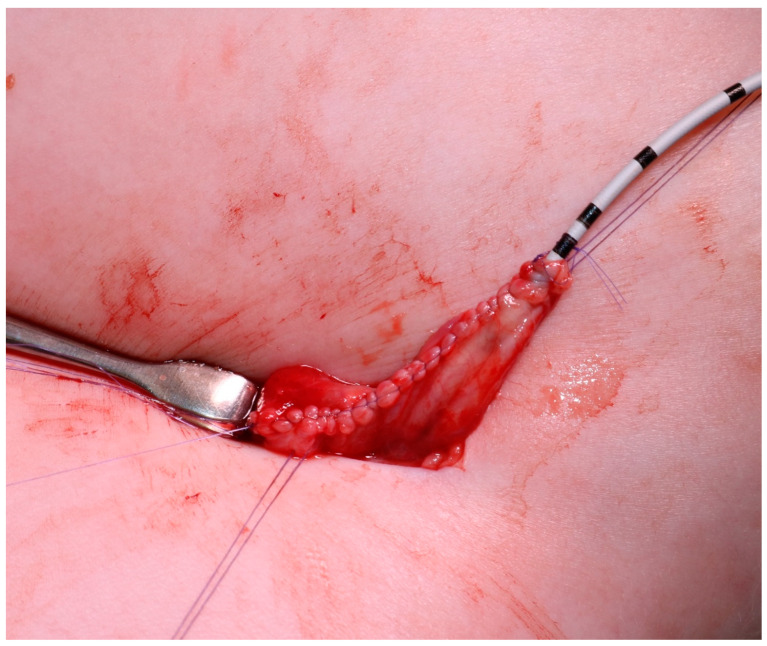
Final aspect of the reconstructed pelvis, with an external ureteropelvic stent emerging from one end of the suture.

The retroperitoneal working space is created through insufflation of CO_2_ (pressure 8–10 mmHg, flow 0.5–1 l/min; according to the patient’s size and weight) and moving the telescope with an Endo peanut; once the lower renal pole is identified, the pelvis and the proximal ureter are anteriorly approached targeting the UPJ. Small vessels are coagulated by unipolar cautery. The uretero-pelvic junction is then isolated with an “L” dissector and exteriorized through lumbar incision, after previously placing a vessel loop for traction purposes. The Anderson-Hynes pyeloplasty is then performed in a traditional fashion, using 6-0 or 7-0 PDS running sutures. The pelvis is repositioned into the renal lodge and the anastomosis can be checked with a retroperitoneoscopic look. A soft Penrose drain is left in place near the anastomosis and the wound is closed by absorbable sutures (Figure 1, Figure 2, Figure 3, Figure 4 and Figure 5). At the beginning of our experience with this approach we used to leave a JJ stent inserted from above before concluding the anastomosis. Because of some mechanical complications with this type of stent, we then chose not to leave any stent, as many authors currently do. After a couple of urinary leakages, at present we prefer using external pyelo-ureteral stents.

Regarding the surgical outcome of the procedure, we considered the minimum period of follow-up to be one year after surgery to define success. Patients with an incomplete follow-up were excluded.

We registered the following data: side of the affected kidney, age at surgery, operative time, length of hospital stay, number and type of complications, number of conversions and data on stenting.

In accordance with literature [11], we evaluated patients with ultrasounds after 3, 6, and 12 months, and then once a year. This timing of follow-up was used as an internal standard protocol. Ultrasounds were not performed and re-scheduled if the infant was not well hydrated A post-operative renogram was performed if significant functional asymmetry was detected on pre-operative renography and/or if pelvic dilatation worsened after surgery; if more than one renogram was performed, we selected the one performed between the first and third year after surgery because in the study we wanted to evaluate “the short–medium outcomes”.

For renal units affected by double congenital urinary malformation such as vesico-ureteral reflux (VUR) and vesico-ureteral obstruction (VUO), surgical resolution of the UJPO was determined by a renogram performed at least 12 months after the last surgical procedure on the urinary tract.

We also evaluated patients by calculating the hydronephrosis score system (HSS), before and 1 year after surgery [12]. This score has the advantage of objectively evaluating hydronephrosis severity, considering the APD, the level of SRF and the entity of the urinary flow with a MAG-3 renal scan. This allowed us to develop an important evaluation of hydronephrosis improvement at 1 year after surgery, comparing the score in that period with the one obtained before treatment. The parameter that mostly contributes in the score is urinary flow; it is considered “obstructed” with a half clearance time (T/2) more than 20 min, “uncertain” between 15–20 min and “good” when less than 15 min. Data were compared using the Student *t* test and chi-square test. *p* < 0.05 defined a statistically significant difference.

## 3. Results

Between January 2005 and September 2018, in our unit, 149 OTAPs were performed by one of the four senior surgeons, assisted by a younger surgeon. Among these cases, we selected the ones with severe hydronephrosis made within the first 60 days of life: 68 patients (57 males and 11 females). We divided these patients into two groups: Group 1 was composed of 34 patients (27 males and 7 females) treated within 90 days of life, while Group 2 was composed of 34 patients (30 males and 4 females) treated after 90 days of life but within the first year of life. Due to lack of follow-up, five patients (14.7%) in Group 1 and seven patients (20.6%) in Group 2 were excluded only from the success rate analysis, but not from the analysis of intraoperative data and early post-operative outcomes.

The mean age at pyeloplasty was 78.24 ± 15.21 days in Group 1 and 186 ± 60.14 days in Group 2.

The mean operative time was 126.68 ± 36.98 min (range 68–200 min) for Group 1 and 134.26 ± 47.04 min (range 55–235 min) for Group 2. There was no statistically significant difference between groups (*p* = 0.81).

One patient (2.94%) in Group 1 and two patients (5.88%) in Group 2 were converted to an open approach, due to an accidental breaking of peritoneum. There was no statistically significant difference between groups (*p* = 0.55).

At the end of the procedure, we left a ureteropelvic stent in 29 patients (21 JJ stents and 8 external uretero-pelvic stent) in Group 1 and 30 patients (11 JJ stents and 19 external uretero-pelvic stent) in Group 2. The mean hospitalization was 7.94 ± 3.43 days (range 4–21 days) in Group I and 7.09 ± 4.81 days (range 3–28 days) in Group II. There was no statistically significant difference between groups (*p* = 0.50).

In Group 2 we registered two early complications (5.88%), consisting of anastomosis dehiscence that determined urinary leakage; one healed conservatively, one needed a second surgical procedure. There was no statistically significant difference between groups (*p* = 0.15).

The number of renal units affected by double congenital urinary malformation (VUR and VUO) was one (2.94%) in Group 1 and one (2.94%) in Group 2; these associated anomalies were surgically corrected at 12 months of age.

Patients’ demographics, and intra- and early post-operative data are summarized in Table 1.

In these patients the mean follow-up was 40.86 ± 32.68 months (range 12–132) in Group 1 and 50.19 ± 34.04 months (range 12–107) in Group 2.

The mean APD before surgery in Group 1 was 34.21 ± 11.67 mm and 32.36 ± 9.71 mm in Group 2. At 1 year after surgery, the mean APD was 16.95 ± 11.48 mm in Group 1 and 14.12 ± 7.76 mm in Group 2. There was no statistically significant difference between groups (*p* = 0.35).

In total, 13 out of 29 patients (44.8%) in Group 1 and 7 out of 27 patients (25.9%) in Group 2 were also evaluated with a post-operative MAG3 renal scan, performed between the first and third years after surgery.

In Group 1, the mean pre-operative SRF was 35.73 ± 11.70%, whereas the mean post-operative SRF was 40.31 ± 9.62% with no significant improvement (*p* = 0.28). In Group 2, the mean pre-operative SRF was 41.50 ± 11.60%, whereas the mean post-operative SRF was 41.67 ± 14.02% with no significant improvement (*p* = 0.98).

We registered one recurrence in Group 1 (3.44%) and two recurrences (7.41%) in Group 2, without statistically significant differences between groups (*p* = 0.48). The results are summarized in Table 2.

**Figure 6:**
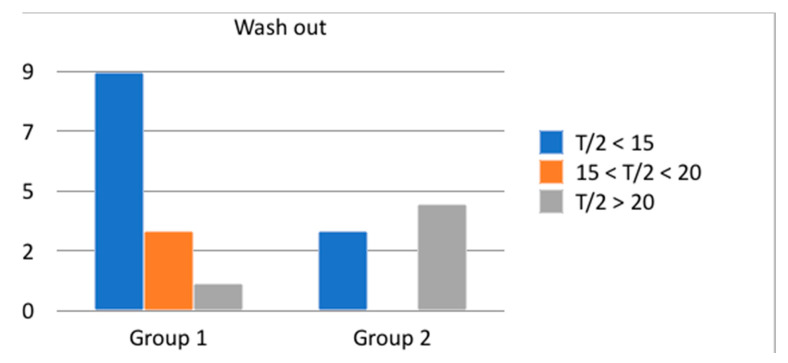
Urinary flow, after surgery, evaluated with renogram (wash-out, expressed with T/2).

Figure 6 represents the post-operative urinary flow of patients, in both groups, expressed with T/2. In Group 1, 9 out of 13 patients expressed a T/2 lower than 15 min, 3 out of 13 patients from 15 to 20 min and only 1 patient of 13 expressed a T/2 more elevated than 20 min. In Group 2, 3 of 7 patients expressed a T/2 lower than 15 min and 4 of 7 patients more elevated than 20 min. The difference between these two groups was statistically significant (*p* = 0.037).

Table 3 represents the “hydronephrosis severity score” (HSS), before and at 1 year after surgery. In Group 1 the mean preoperative HSS of 9.08 ± 1.56 improved to a mean post-operative HSS of 4.42 ± 3.29. In Group 2 the mean preoperative HSS of 8.71 ± 2.14 slightly improved to a mean post-operative HSS of 6.43 ± 2.82. In fact, in Group 1, the mean postoperative reduction in HSS was 4.67 ± 2.23, meaning a good improvement. In Group 2, the mean postoperative reduction in HSS was 2.29 ± 1.50, meaning a mild improvement. The difference between these two groups was statistically significant (*p* = 0.023). Thus, thanks to this score we registered an important improvement in patients treated earlier, which did not happen for delayed pyeloplasty.

## 4. Discussion

The ureteropelvic junction (UPJ) is the most common site of obstruction in the upper urinary tract during the pediatric age.

Diagnosis is often made in the first months of life, it being a confirmation of a prenatal diagnostic suspicion.

The only possible therapy for a real UPJ stenosis is surgical correction in order to create a normal urinary flow and preserve residual renal function.

Since 1949 [13], the year of first description, the gold standard for the correction of UPJO has been the dismembered pyeloplasty described by Anderson-Hynes. This technique has evolved throughout time from an initial open approach to others less invasive.

While it is already well established that a symptomatic patient should be immediately treated surgically [3], unfortunately, to date, there is no univocal consensus about the timing of surgical intervention and the appropriate technique for asymptomatic neonates and infants, despite the presence of thousands of studies in the literature concerning UPJO.

One of the main concerns is the risk of further renal damage with consequent loss of renal function. In this respect, Chertin et al. recommend an initial conservative management, because, even if there is a loss of SRF, it can easily be recovered with a delayed surgical correction [7]. Babu et al., at odds, reported how a delayed surgical reconstruction of UPJO can cause an irreversible loss of renal function, avoidable by early intervention [4]. In particular, early pyeloplasty would lead to significant improvement of SRF at 1-year follow-up, while delayed pyeloplasty would lead to a marginal but significant loss.

Jiang et al. suggest performing an early pyeloplasty in patients with prenatally diagnosed SFU grades 3–4 UPJO because prolonged waiting might affect renal functional recovery [14]. Tabari et al., in the same way, showed how an early intervention induces an improvement in anatomic and functional values, while a delayed correction of UPJO implicates a major risk of renal function deterioration [15].

It is well known how minimal invasive approaches allow a reduction of post-operative hospitalization, pain and scars [16]. The major impediment for laparoscopic (LPSc) and retroperitoneoscopic (RPSc) approaches in those patients concerns the small operative space and technical difficulties that increase the risk of abdominal injuries [17,18]. For these reasons, some authors suggest to not perform pyeloplasty laparoscopically in young infants [19,20].

Since 2005 the preferred approach, in our institution, for hydronephrosis in children younger than 2 years has been the one-trocar-assisted pyeloplasty (OTAP).

We have already demonstrated this technique to be feasible in terms of efficacy and safety in the first 90 days of life [10]. OTAP puts together the advantages of minimally invasive surgery and traditional dismembered pyeloplasty by Anderson-Hynes, and is therefore an excellent option for treating this category of patients [8,9].

We analyzed our series of patients, selecting those who were born with the same condition (see criteria above) but treated in different periods; this allowed us to investigate the possible presence of intraoperative differences and postoperative outcomes. We analyzed 68 patients, divided in two groups: one made of 34 patients treated before 90 days of life, the other of the same number but treated after 90 days of life.

The indications we chose for early surgical correction were verified within 60 days of life in both groups: APD > 20 mm, and an obstructive pattern on MAG3 with furosemide associated with an impaired echotexture and/or separate renal function (SRF) < 40%.

No significant differences have been found intraoperatively between groups. The mean operative time, conversions and complications were slightly greater in the patients treated after 90 days of life. This result demonstrates that the small working spaces do not affect the procedure.

We evaluated these patients at 1 year after surgery with the HSS [12]. This score has the advantage of objectively evaluating hydronephrosis severity, considering the APD, the level of SRF and the entity of the urinary flow with a MAG-3 renal scan. This allowed us to perform an important evaluation of hydronephrosis improvement at 1 year after surgery, comparing the score in that period with the one obtained before treatment. Thanks to this score we registered an important improvement in patients treated earlier, which did not happen for delayed pyeloplasty. Nevertheless, HSS is not a functional score but only a morphological description. It is evident then an improvement in HSS does not necessarily mean a physiological benefit to patients, so it may be only a partial consideration in the management of patients with UJPO.

Our study has some limitations. Data were retrospectively analyzed and this could lead to errors; with the preliminary results obtained thus far with this retrospective analysis, we are about to start a prospective study with the same intention, in order to verify what has been said. Over a period of 15 years, we have gained experience with OTAP. In fact, the conversions and some of the complications reported happened in the first 8 years of experience. This might represent another bias. Furthermore, only selected patients are evaluated post-operatively with renography, thus the number of these patients is not very high, though sufficient to be statistically significant. The study cannot definitely establish the superiority of an early timing of surgical correction, but it is evident that further research is needed to clarify this aspect. Our future prospective study presents this goal.

## 5. Conclusions

As claimed in the previous study, we suggest the OTAP technique as a suitable option for the treatment of neonates and infants affected by UPJO, in the first 90 days of life. In our study, there was no significant evidence, in terms of intraoperative data and early postoperative outcomes, between patients who underwent an early pyeloplasty and those who underwent a delayed correction. Moreover, we registered a significant improvement in those patients with an impaired SRF that underwent an early surgical correction, especially in terms of urinary flow. We hope to confirm in the future our preliminary results obtained in this work.

## Figures and Tables

**Table 1:** Patients’ demographics, and intra- and early post-operative data.

	GROUP I (*n* = 34)	GROUP II (*n* = 34)	*p*
**Age at surgery, days (range)**	78.24 ± 15.21 (35–90)	186 ± 60.14 (125–418)	
**Side**			
Right (%)	13 (38.2%)	9 (26.5%)	
Left (%)	21 (61.8%)	25 (73.5%)	
**Mean operative time, min (range)**	126.68 ± 36.98 (60–200)	134.26 ± 47.04 (55–235)	0.81
**Mean hospitalization, days (range)**	7.94 ± 3.43 (4–21)	7.09 ± 4.81 (3–21)	0.50
**Complications**			
Early complications (%)	0	2 (5.88 %)	0.15
**Conversion**	1 (2.94%)	2 (5.88%)	0.55
**Stent**			
No stent (%)	5 (14.17%)	4 (11.8%)	
JJ	21 (61.8%)	11 (32.4%)	
External	8 (23.5%)	19 (55.9%)	

**Table 2:** Results.

	GROUP I	GROUP II	*p*
**AP Diameter (APD)**			
Initial APD, mm	34.21 ± 11.67	32.36 ± 9.71	
APD before surgery, mm	-	27.75 ± 8.40	
APD at 1 year after surgery, mm	16.95 ± 11.48	14.12 ± 7.76	
Final ADP outcome, mm	18.34 ± 1.37	18.25 ± 9.59	0.35
**Split Renal Function (SRF)**			
Mean SRF within 60 days of life %	37.9 ± 10.67	43.7 ± 12.51	
**Complications**			
Recurrence (%)	1 (3.70 %)	2 (8.33 %)	0.48
**Mean Follow-up, months (range)**	40.86 ± 32.68 (12–132)	50.19 ± 34.04 (12–107)	

**Table 3:** “Hydronephrosis severity score” (HSS), before and at 1 year after surgery.

	GROUP I (*n* = 14)	GROUP II (*n* = 7)	*p*
**Split Renal Function (SRF)**			
Mean SRF before surgery, %	35.73 ± 11.70	41.50 ± 11.60	
Mean SRF at 1 year after surgery, %	40.31 ± 9.62	41.67 ± 14.02	0.28 (I)–0.98 (II)
**Hydronephrosis Severity Score (HSS)**			
HSS before surgery	9.08 ± 1.56	8.71 ± 2.14	
HSS after surgery	4.42 ± 3.29	6.43 ± 2.82	
HSS improvement	4.67 ± 2.23	2.29 ± 1.50	0.02
